# Kappa-Opioid Receptor Signaling in the Striatum as a Potential Modulator of Dopamine Transmission in Cocaine Dependence

**DOI:** 10.3389/fpsyt.2013.00044

**Published:** 2013-06-03

**Authors:** Pierre Trifilieff, Diana Martinez

**Affiliations:** ^1^New York State Psychiatric Institute, Columbia University, New York, NY, USA; ^2^NutriNeuro, UMR 1286 INRA, University Bordeaux 2, Bordeaux, France

**Keywords:** imaging, kappa opioid receptor, dopamine, cocaine dependence, striatum, dopamine receptor

## Abstract

Cocaine addiction is accompanied by a decrease in striatal dopamine signaling, measured as a decrease in dopamine D2 receptor binding as well as blunted dopamine release in the striatum. These alterations in dopamine transmission have clinical relevance, and have been shown to correlate with cocaine-seeking behavior and response to treatment for cocaine dependence. However, the mechanisms contributing to the hypodopaminergic state in cocaine addiction remain unknown. Here we review the positron emission tomography (PET) imaging studies showing alterations in D2 receptor binding potential and dopamine transmission in cocaine abusers and their significance in cocaine-seeking behavior. Based on animal and human studies, we propose that the kappa receptor/dynorphin system, because of its impact on dopamine transmission and upregulation following cocaine exposure, could contribute to the hypodopaminergic state reported in cocaine addiction, and could thus be a relevant target for treatment development.

## Introduction

Studies imaging the neurochemistry associated with cocaine addiction in humans have largely focused on dopamine signaling in the striatum. These studies show that pre-synaptic dopamine release, in response to the administration of a stimulant, is reduced in cocaine abusers compared to healthy controls. This has important implications for this disorder, since the reduction in dopamine release has been shown to correlate with increased cocaine-seeking behavior. Importantly, the imaging studies were performed at about 14 days abstinence, which has clinical relevance, since previous studies have shown that cocaine abusers who achieve 2 weeks of abstinence have a better treatment response compared to those who do not (Bisaga et al., 2010; Oliveto et al., 2012). Thus, a better understanding of the mechanisms behind blunted dopamine release would be expected to have implications for treatment development. Among the possible mechanisms that are known to regulate striatal dopamine release is dynorphin acting at the kappa receptor. Kappa receptor activation in the striatum has been shown to inhibit stimulant-induced dopamine release, in addition to striatal dopamine levels and dopamine neurons activity (for review, see Koob and Le Moal, 2008; Muschamp and Carlezon, 2013). Furthermore, studies in humans and animals show that dynorphin is significantly upregulated following chronic cocaine exposure, and that this effect is long lasting (for review, see Koob and Le Moal, 2008; Muschamp and Carlezon, 2013), which could account for the decrease in dopamine signaling seen after 2 weeks of abstinence in the human imaging studies. Here, we review the data suggesting that the cocaine-induced elevation in dynorphin may contribute to the hypodopaminergic state observed in cocaine addiction.

## PET Imaging of Dopamine Transmission in Cocaine Addiction

### Principles of PET imaging

**Figure 1:**
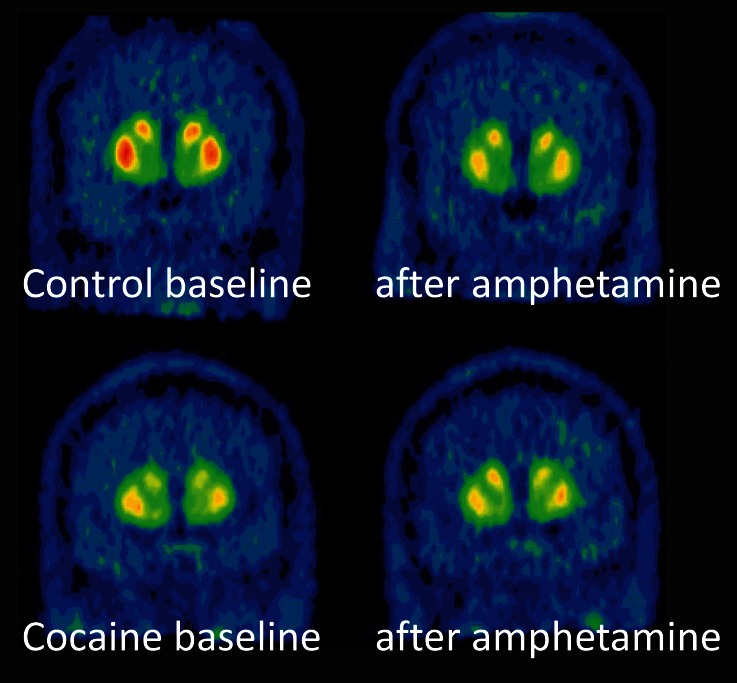
**PET scans in a healthy control and cocaine-dependent subject**. The comparison of the top panels (pre- and post-amphetamine administration) in the healthy control shows that radiotracer ([11C]raclopride) binding is reduced in the striatum following amphetamine. The cocaine-dependent subject (bottom panel) has have lower D2 receptors compared the control in the baseline condition. In addition, the cocaine abuses has less radiotracer displacement (Δ BPND) following amphetamine. Adapted from Trifilieff and Martinez “Cocaine: Mechanism and Effects in the Brain” in “The Effects of Drug Abuse on the Human Nervous System” M. Kuhar and B. Madras editors, 2012, publisher Neuroscience-Net, LLC.

Positron emission tomography (PET) allows imaging of the neurochemistry associated with drug and alcohol addiction in the human brain. This imaging modality uses radionuclide-labeled ligands that bind to a specific receptor, and the radioligands used most frequently in addiction research label the dopamine receptors. Radiotracers that label the dopamine type 2 family of receptors (referred to as D2) can also be used to measure changes in extracellular dopamine. This is performed by imaging with radiotracers that are sensitive to changes in extracellular dopamine, and obtaining scans before and after the administration of a psychostimulant (such as amphetamine or methylphenidate). These stimulants increase extracellular dopamine levels, which results in a reduction of dopamine receptors that are available to bind to the radiotracer, shown in Figure 1. For reasons that are not completely understood, this method can be used with most D2 receptor radiotracers but not with radiotracers that bind to the D1 receptor. Thus, imaging studies using the D2 receptor radiotracers (such as [11C]raclopride or [18F]fallypride) can be used to measure changes in endogenous dopamine, whereas radiotracers that label the D1 receptor (such as [11C]NNC112 or [11C]SCH23390) cannot (Abi-Dargham et al., 1999; Chou et al., 1999; Laruelle, 2000; Martinez and Narendran, 2010).

The main outcome measure in radioligand imaging studies is receptor binding to the radiotracer, referred to as BPND, defined as the ratio of specific to non-specific binding (Innis et al., 2007). The change in extracellular dopamine resulting from stimulant administration is measured by comparing baseline BPND (pre-stimulant administration) and BPND following the stimulant. This is used to derive the percent change in BPND, or ΔBPND, defined as [(BPNDbaseline – BPNDchallenge)/BPNDbaseline]. Previous studies in non-human primates have shown that ΔBPND correlates linearly with changes in extracellular dopamine, measured with microdialysis (Breier et al., 1997; Endres et al., 1997; Laruelle et al., 1997). Thus, ΔBPND provides an indirect measure of stimulant-induced pre-synaptic dopamine release, and can be used to characterize the alterations in dopamine signaling that occur in cocaine dependence.

### PET imaging of dopamine receptors in cocaine addiction

To date, six studies have been performed imaging the D2 receptor in cocaine abusers, and these consistently show a decrease in binding in the striatum compared to matched controls (Volkow et al., 1990, 1993, 1997; Martinez et al., 2004, 2009a, 2011). The decrease is about 15–20% and occurs in both the ventral and dorsal striatum. Importantly, animals with low D2 receptor levels in the striatum, prior to drug exposure, display greater cocaine self-administration (Morgan et al., 2002; Czoty et al., 2004; Nader et al., 2006; Dalley et al., 2007). Imaging studies in humans show that low striatal D2 receptor binding in cocaine abusers in the striatum correlates with decreases in glucose metabolism in the orbito-frontal cortex and cingulate gyrus, which process drive and affect, and may lead to continued drug-taking behavior (Volkow et al., 1993, 1999). Several authors have proposed that changes in D2 receptor binding in addiction could reflect behavioral vulnerability to drug self-administration, such as lack of cognitive control or increased impulsivity (Everitt et al., 2008; Dalley et al., 2011; Groman and Jentsch, 2012).

One PET imaging study has measured D1 receptor binding in cocaine abuse (Martinez et al., 2009b). This study showed no difference in D1 receptor binding in cocaine abusers compared to controls, which is consistent with a post-mortem study of striatal D1 receptor mRNA (Meador-Woodruff et al., 1993). However, the imaging study also showed that, within the cocaine-dependent subjects, low D1 receptor binding in the ventral striatum was associated with greater choices to self-administer cocaine. Thus, this finding may represent a phenotype in which low D1 receptor binding in the limbic striatum is associated with a greater vulnerability to the reinforcing effects of cocaine. This is in agreement with pharmacologic studies in humans showing that stimulation of D1 receptors reduces, whereas blockade of the D1 receptor enhances, the reinforcing effects of cocaine (Haney et al., 1999, 2001). Taken together, these studies indicate that decreased signaling at the D1 receptor may be associated with more cocaine-taking behavior.

### PET imaging dopamine release in cocaine abusers

Imaging studies measuring pre-synaptic dopamine release show that cocaine dependence is associated with a reduction in responsiveness of the dopamine system to a stimulant challenge. For example, in healthy human volunteers, the administration of a psychostimulant produces a decrease in [11C]raclopride binding (ΔBPND) of 15–20% (Volkow et al., 1994; Drevets et al., 2001; Martinez et al., 2003; Munro et al., 2006), but in cocaine abusers the decrease in [11C]raclopride binding is significantly blunted (Volkow et al., 1997; Malison et al., 1999; Martinez et al., 2007b, 2011). Thus, four studies have shown that cocaine dependence is associated with reduced [11C]raclopride displacement following stimulant administration compared to healthy controls, which represents a reduction in pre-synaptic dopamine release. PET imaging studies also show that cocaine abuse is associated with both decreased [18F]DOPA uptake and striatal vesicular monoamine transporter 2 binding, which provide measures of pre-synaptic dopamine stores (Wu et al., 1997; Narendran et al., 2012).

In addition to a reduction in stimulant-induced dopamine release, PET imaging has also shown that dopamine levels in the resting condition (without any stimulant administration) are reduced in cocaine dependence. This is performed by imaging the D2 receptors before and after acute depletion of endogenous dopamine using alpha-methyl-para-tyrosine (AMPT). Thus, imaging after AMPT administration results in an increase in [11C]raclopride binding, as opposed to the decrease seen after stimulant administration (Martinez et al., 2009a). AMPT administration resulted in an increase of 11.1 ± 4.4% in [11C]raclopride binding in the striatum for healthy controls, but only 5.7 ± 5.9% for cocaine-dependent volunteers (Martinez et al., 2009a), indicating that basal dopamine levels are decreased in cocaine abuse.

Taken together, imaging studies in cocaine abuse consistently show a reduction in striatal dopamine transmission, compared to healthy controls, measured as decreased pre-synaptic dopamine release (Volkow et al., 1997; Malison et al., 1999; Martinez et al., 2007b, 2011) and reduced baseline levels of endogenous dopamine (Martinez et al., 2009a). Similar findings have been shown in rodents (Parsons et al., 1991; Robertson et al., 1991; Rossetti et al., 1992; Weiss et al., 1992; Gerrits et al., 2002) and non-human primates (Castner et al., 2000; Kirkland Henry et al., 2009). Thus, cocaine dependence is associated with a hypodopaminergic state, which correlates with behaviors that contribute to addiction and relapse (Melis et al., 2005). Importantly, the PET scans showing blunted dopamine release were obtained after about 2 weeks of abstinence, to avoid the acute effect of cocaine on dopamine signaling, and due to the clinical relevance of this time point. Previous studies have shown that cocaine abusers who can achieve 2 weeks of abstinence have a better treatment response compared to those who do not (Bisaga et al., 2010; Oliveto et al., 2012).

## Significance of the Hypodopaminergic State in Cocaine Abuse

The impact of dopamine transmission on addiction has been demonstrated for decades, but its actual role in mediating the reinforcing effects of drugs of abuse remains under debate. Dopamine does not appear to only signal “reward” (drug or natural rewards), although dopamine neurons fire in response to the receipt of a reward, and during the expectation of a reward. However, dopamine signaling more likely mediates the reinforcing effects of natural rewards and abused drugs, and makes the behavior required to obtain the reward more likely to be repeated (Schultz, 2006; Berridge, 2007; Wise, 2008; Salamone and Correa, 2012). However, the imaging studies in cocaine dependence consistently show that pre-synaptic dopamine is reduced compared to controls, indicating that this disorder is associated with a hypodopaminergic state. This plays a crucial role in drug-seeking and taking, even after prolonged drug-free periods (Melis et al., 2005).

The imaging studies in human cocaine abusers show that blunted dopamine release correlates with an increase in cocaine self-administration (Martinez et al., 2007b, 2011). These studies showed that low dopamine release in cocaine abusers, measured as ΔBPND, was associated with the decision to take cocaine in the presence of competing non-drug reinforcers. The inability of the cocaine-dependent subjects with low dopamine release to alter their behavior can be viewed as an inability to respond to alternative sources of reward. This is consistent with the theory that decreased dopamine function in addiction results in a decreased interest to non-drug-related stimuli and increased susceptibility to the drug of choice (Melis et al., 2005).

These studies raise the question regarding the mechanism behind this decrease in pre-synaptic dopamine release. Previous studies in animals have shown that cocaine exposure results in reduced burst firing of the dopamine neurons of the ventral tegmental area (Brodie and Dunwiddie, 1990; Lacey et al., 1990; Ackerman and White, 1992; Gao et al., 1998). Decreases in extracellular dopamine levels in the nucleus accumbens have also been reported following cocaine withdrawal (Parsons et al., 1991; Robertson et al., 1991; Rossetti et al., 1992; Weiss et al., 1992). Cocaine administration has also been shown to alter the sensitivity of D2 autoreceptors of the midbrain (Gao et al., 1998; Lee et al., 1999; Marinelli et al., 2003), which could reduce pre-synaptic dopamine release. In addition to these functional changes in dopamine signaling, animal studies have also shown that cocaine exposure produces morphological changes in dopamine neurons. These include alterations in dendritic spine density and morphology and a reduction in the size of the dopamine neurons of the ventral tegmental area (Melis et al., 2005).

Presently, it is unknown whether these changes occur in the human brain. Human studies of the dopamine transporter (DAT), which can serve as a marker for the integrity of the dopamine neurons (Fusar-Poli and Meyer-Lindenberg, 2013), show that the DAT is increased in post-mortem studies of cocaine abusers (Little et al., 1993, 1999). However, imaging studies show that the DAT is increased for a short time period following the cessation of cocaine use, but soon return to control levels (Volkow et al., 1996; Wang et al., 1997; Malison et al., 1998). But measuring DAT binding alone is unlikely to reveal morphological alterations of the dopamine neurons, and other means for investigating this with imaging in humans are not yet available. With respect to the dopamine receptors in the midbrain, one study in methamphetamine abusers and another in cocaine abusers showed that D3 receptor binding is elevated in the substantia nigra/ventral tegmental area (SN/VTA) compared to controls (Matuskey et al., 2011; Boileau et al., 2012). The specific role of the D3 receptor in the modulation of dopamine transmission and its function as an autoreceptor are still highly debated (Sokoloff et al., 2006). However, considering the possible implication of this receptor in modulating dopamine synthesis and release (for review, Gross and Drescher, 2012), an increase in D3 receptor levels in SN/VTA may contribute to the hypodopaminergic state observed in addiction.

In addition to alterations in the dopamine neurons themselves, it is possible that other neurotransmitter systems may be regulating the dopamine system. Candidates include the glutamatergic, GABAergic, serotoninergic, or noradrenergic afferents to the dopamine and striatal neurons, which have been reviewed previously (Melis et al., 2005; Gerfen and Surmeier, 2011). In this review, we focus on the kappa/dynorphin system as a potential modulator of dopamine release in cocaine abuse for the following reasons: (1) among the neurotransmitters that modulate dopamine transmission, evidence from human and animal studies show that cocaine exposure significantly upregulates kappa/dynorphin signaling (for review, see Wee and Koob, 2010; Muschamp and Carlezon, 2013); (2) in the striatum, dynorphin signaling strongly regulates dopamine signaling and animal studies show that activation of the kappa system reduces pre-synaptic dopamine release (Koob and Le Moal, 2008; Muschamp and Carlezon, 2013). Thus, elevated striatal dynorphin activity at the kappa receptor could be a compensatory adaptation that inhibits psychostimulant-induced dopamine release (Koob and Le Moal, 2008; Muschamp and Carlezon, 2013).

## Dynorphin and Kappa Receptors

### Kappa receptor/dynorphin signaling

Dynorphin (DYN) is the class of peptides cleaved from prodynorphin, which include dynorphin A and B (and others) which have a high affinity for the kappa receptor (KOR) (Chen et al., 2007). Currently, only one KOR subtype (type 1) has been cloned, and while types 2 and 3 have been hypothesized, they have yet to be fully characterized (Shippenberg et al., 2007). KOR selective agonists and antagonists have been developed in recent years, allowing investigation into the neurochemical and behavioral effects of the DYN/KOR system. The KOR agonists include the arylacetamides U69593 and U50488, and salvinorin A, a naturally occurring alkaloid found in the plant *Salvia divinorum* (Von Voigtlander and Lewis, 1982; Lahti et al., 1985; Roth et al., 2002). The selective KOR antagonists include nor-binaltorphimine (nor-BNI), 5′-guanidinonaltrindole (GNTI), and JDTic (Endoh et al., 1992; Jones and Portoghese, 2000; Carroll et al., 2004). Activation of the KOR is aversive in both humans and animals, and KOR agonists are not self-administered by animals (Mucha and Herz, 1985; Tang and Collins, 1985; Pfeiffer et al., 1986; Bals-Kubik et al., 1993; Walsh et al., 2001; Wadenberg, 2003), although the same cannot be said of some humans.

KOR signaling is complex and agonists have been shown to activate, inhibit and/or have no effect on downstream signaling (i.e., cAMP, IP3/DAG, and Ca^2+^) depending on experimental conditions (Tejeda et al., 2012). It is likely that KOR agonists display inverted *U*-shape effects, because of KOR ability to recruit both inhibitory Gβγ, Gα_i_, Gα_o_, Gα_z_, and Gα_16_, and stimulatory, Gα_s_, G-proteins (Law et al., 2000; Tejeda et al., 2012). Nanomolar ligand concentrations result in the recruitment of inhibitory G-proteins and a decrease in membrane excitability as well as transmitter release via stimulation of K^+^-channel activity (Grudt and Williams, 1993) and inhibition of Ca^2+^-channel and pre-synaptic release machinery activity (Gross et al., 1990; Iremonger and Bains, 2009). In contrast, sub-nanomolar ligand concentrations may result in coupling of KOR to Gαs and produce opposite effects (Crain and Shen, 1996; Tejeda et al., 2012). It should be noted that KOR activity can modulate D2 autoreceptor-dependent decrease in dopamine release by signaling interaction (Jackisch et al., 1994; Acri et al., 2001; Fuentealba et al., 2006).

### Kappa receptor/dynorphin in direct and indirect pathways of the striatum

The medium spiny neurons (MSNs) can be categorized into at least two subgroups according to their projections sites and the proteins they express (Gerfen, 2000; Gerfen and Surmeier, 2011). The “direct” or striatonigral pathway made up of MSNs that project monosynaptically to the medial globus pallidus and back to the dopamine neuron cell bodies of the substantia nigra. MSNs from the direct pathway express the dopaminergic D1 receptor, M4 muscarinic acetylcholine receptor, substance P, and dynorphin. The indirect striatopallidal pathway is composed of MSNs that project to the lateral globus pallidus, which reach the substantia nigra through synaptic relays through the lateral globus pallidus and subthalamic nucleus. These MSNs express the dopaminergic D2 receptor, adenosine receptors and enkephalin. It should be noted that the segregation of these two populations of MSNs has been established in the dorsal striatum, but that several studies show that a subpopulation of MSNs in the NAc seem to co-express D1 and D2 receptors (George and O’Dowd, 2007; Valjent et al., 2009). Dopamine can activate or inhibit cyclic AMP-dependent signaling through D1 receptor and D2 receptor respectively, as we will review below. Therefore, dopamine is likely to have differential effects on D1- and D2-expressing MSNs and recent data suggest that, cocaine administration activate signaling pathways in D1-expressing, but actively inhibits them in D2-expressing MSNs (McClung et al., 2004; Bateup et al., 2010), which could account for the imbalance between direct and indirect pathways in addiction (Lobo et al., 2010; Pascoli et al., 2012).

**Figure 2:**
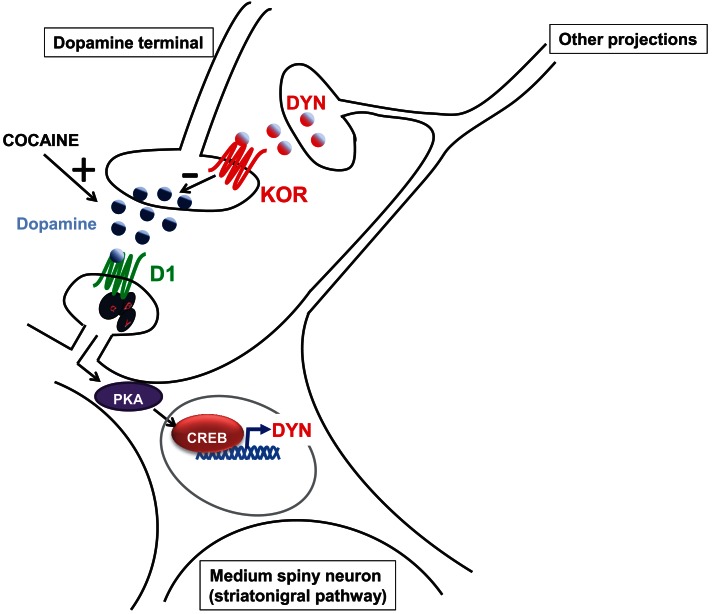
**Model by which the dynorphin/kappa system could counteract cocaine-induced dopamine release**. Cocaine administration elevates dopamine levels. Binding of dopamine on the D1 receptor expressed by medium spiny neurons from the striatonigral pathway (direct pathway) activates the cAMP/PKA/CREB pathway which leads to dynorphin (DYN) synthesis. Binding of DYN on kappa receptors (KOR) located on dopamine terminals exert an inhibitory effect on dopamine release.

D1 receptors recruit adenylyl cyclase through activation of the stimulatory Gα_s_ protein and consequently stimulate the production of adenosine 3′, 5′-monophosphate (cAMP) which leads to the activation of protein kinase A (PKA)-dependent signaling pathways. In contrast, D2 receptor inhibits adenylyl cyclase and cAMP/PKA pathways by recruiting inhibitory Gα_i_. Accordingly, cocaine activates PKA signaling pathway mainly through activation of D1 receptor and manipulation of this pathway alters behavioral responses to cocaine (Girault, 2012). One of the downstream targets of PKA is the transcription factor CREB. Interestingly, whereas overexpression of CREB in the nucleus accumbens reduces the rewarding properties of cocaine, overexpression of a dominant-negative form enhances it (Carlezon et al., 1998; Walters and Blendy, 2001; McClung and Nestler, 2008) suggesting that activation of CREB could counteract the postsynaptic effects of cocaine and therefore decrease behavioral response to cocaine. One of the downstream genes regulated by CREB in the nucleus accumbens encodes preprodynorphin, the precursor gene product of dynorphin (McClung and Nestler, 2008). Activation of the kappa receptor decreases cocaine-induced dopamine release (for review, see Wee and Koob, 2010; Muschamp and Carlezon, 2013). Accordingly, stimulation of the D1 receptor elevates dynorphin expression, which can be blocked with receptor antagonists (Liu and Graybiel, 1998). Thus, it has been proposed that activation of the D1/PKA/CREB pathway could be counteracting the effects of cocaine through synthesis and release of dynorphin (for review, see Wee and Koob, 2010; Muschamp and Carlezon, 2013), shown in Figure 2.

### Kappa receptor/dynorphin and dopamine signaling

The DYN/KOR receptor system has been shown to play a significant role in regulating striatal dopamine transmission. DYN immunoreactive axon terminals originating from D1 receptor-expressing MSNs are found in the caudate, putamen, and nucleus accumbens (Hurd and Herkenham, 1995; Van Bockstaele et al., 1995). The KOR is expressed both pre- and post-synaptically on dopamine neurons, and the pre-synaptic KOR is apposed to DAT on the dopamine axon terminals, indicating that this system closely regulates the mesoaccumbal dopamine neurons (Svingos et al., 2001).

A number of animal studies have shown that the administration of a KOR agonist reduces dopamine levels in the striatum and dopamine neuron activity in the nucleus accumbens and ventral tegmental area (Di Chiara and Imperato, 1988; Heijna et al., 1990, 1992; Donzanti et al., 1992; Spanagel et al., 1992; Maisonneuve et al., 1994; Xi et al., 1998; Thompson et al., 2000; Margolis et al., 2003; Zhang et al., 2004b). In fact, KOR activation reduces basal dopamine levels as well as stimulant-induced dopamine release (cocaine) (Spanagel et al., 1990; Maisonneuve et al., 1994; Carlezon et al., 2006; Gehrke et al., 2008). Reverse dialysis into the nucleus accumbens reduces extracellular dopamine (Donzanti et al., 1992; Zhang et al., 2004a). Notably, this effect is seen when the KOR agonist is administered into the striatum, whereas administration into the VTA appears to be species dependent (Spanagel et al., 1992; Chefer et al., 2005; Ford et al., 2006; Margolis et al., 2006).

KOR activation has been shown to inhibit electrically evoked [^3^H]dopamine release in the nucleus accumbens (Heijna et al., 1992; Yokoo et al., 1992), which also shows that activation of this receptor reduces striatal dopamine transmission. More recently, Chefer et al. (2005) showed that the deletion of KOR is associated with an enhancement of basal dopamine release. Alternatively, KOR antagonists stimulate the release of dopamine in the striatum (Maisonneuve et al., 1994; You et al., 1999; Beardsley et al., 2005). Lastly, repeated KOR agonist administration reduces striatal D2 receptor density (Izenwasser et al., 1998). These findings show that DYN/KOR signaling exerts inhibitory control over dopamine release and dopamine receptor signaling in the striatum (Bruijnzeel, 2009; Wee and Koob, 2010) and demonstrate that excessive KOR activation significantly reduces striatal dopamine transmission, independent of the modality used to measure dopamine transmission.

Notably, imaging studies show that, in addition to cocaine dependence, addiction to other substances of abuse also results in blunted pre-synaptic dopamine release, measured with PET. This finding has also been reported in studies of alcohol, methamphetamine, opiate, and tobacco dependence (Martinez et al., 2007a, 2012; Busto et al., 2009; Wang et al., 2012). While some studies have shown that the DYN/KOR system plays a role in these disorders as well (for review, see Wee and Koob, 2010; Koob, 2013), the effect of drug exposure on KOR and DYN is less clear and may even be down-regulated in methamphetamine and opiate dependence (Drakenberg et al., 2006; Frankel et al., 2007). Further studies are needed to clarify the interaction between the DYN/KOR system and dopamine signaling in these disorders.

## Kappa Receptor/Dynorphin System in Cocaine Abuse

Three post-mortem studies have been performed investigating KOR binding in cocaine abuse. The first of these, by Hurd and Herkenham (1993), showed a twofold increase in KOR binding in the caudate, but not the putamen or ventral striatum, in cocaine-dependent subjects compared to control subjects. Mash and Staley (1999) used *in vitro* autoradiography and ligand binding to map KOR in the brains of cocaine abusers and showed a twofold increase in the anterior and ventral sectors of the caudate and putamen, and nucleus accumbens compared to controls. Similar results were reported by Staley et al. (1997) who used radiolabeling to measure the KOR and reported a significant increase in KOR in the caudate, putamen, and nucleus accumbens in cocaine exposed compared to control brain tissue. These studies demonstrate that cocaine abuse or dependence is associated with a significant upregulation of the KOR in the striatum. However, to date, no human *in vivo* imaging studies of the KOR have been published in cocaine abuse. While previous PET studies imaged the mu opioid receptor in cocaine dependence (Zubieta et al., 1996; Gorelick et al., 2008), PET imaging of the KOR has not been previously possible due to the lack of an appropriate radiotracer. Therefore, correlations with clinical outcomes, such as cocaine-seeking behavior could not be performed. In addition, these post-mortem studies did not measure markers of dopamine transmission (such as receptor density or dopamine levels), so that it remains unknown whether the increase in KOR signaling coincides with a reduction in dopamine signaling largely described in PET imaging studies. Measuring both KOR binding and dopamine transmission in the same individuals will require the development of new radiotracers for KOR.

### Cocaine administration and dynorphin

A number of animal studies have shown that repeated cocaine administration increases levels of DYN, prodynorphin mRNA, and preprodynorphin mRNA. The initial studies measured peptide levels and showed that chronic dosing of cocaine increased striatal dynorphin levels by 40–100% (Sivam, 1989; Smiley et al., 1990). Further studies measuring prodynorphin and preprodynorphin mRNA, instead of peptide levels, have replicated these findings. Daunais et al. (Daunais et al., 1993, 1995; Daunais and McGinty, 1995, 1996) showed that cocaine self-administration increases preprodynorphin mRNA in the caudate/putamen by more than 100%. Similar results have been reported in studies by other groups as well, where the administration of cocaine has been shown to increase preprodynorphin mRNA levels 50–100% in the caudate/putamen of rats and mice (Yuferov et al., 2001; Zhou et al., 2002; Jenab et al., 2003; Schlussman et al., 2003, 2005; Zhang et al., 2013). Spangler et al. (1993, 1996) demonstrated that cocaine increased prodynorphin mRNA in the caudate/putamen by 40%, and that these levels remained elevated for days. Overall, the above studies in rodents consistently report that cocaine administration increases DYN, prodynorphin, and preprodynorphin mRNA with levels ranging from about 40 to 100%. Previous studies have shown that the levels of DYN peptide and prodynorphin/preprodynorphin mRNAs correlate with each other, suggesting that increases in mRNAs closely reflect increases in the peptide itself (Li et al., 1988; Sivam, 1996).

These findings in rodents have been replicated in studies of rhesus monkeys and humans. Fagergren et al. (2003) performed a study in rhesus monkeys who self-administered cocaine and showed that prodynorphin mRNA levels were increased in the dorsolateral caudate (83%), central caudate (34%), and the dorsal putamen (194%). In humans, Hurd and Herkenham (1993) first reported that cocaine abuse was associated with an increase in preprodynorphin mRNA in the putamen and caudate in a post-mortem study of cocaine abusing subjects compared to control subjects. More recently, Frankel et al. (2008) measured DYN peptide levels in a post-mortem study of cocaine abusers and controls subjects, and reported a significant increase in DYN in the caudate and a trend toward a significant increase in the putamen compared to control subjects. A very large increase was seen in the ventral pallidum but no difference was seen in the thalamus, frontal, temporal, parietal, and occipital cortices. Taken together, these studies indicate that cocaine exposure increases striatal DYN signaling at the kappa receptor in rodents, non-human primates, and humans. Considering the effect of DYN on dopamine signaling, it is likely that the sustained increase in DYN levels by cocaine exposure participates to the hypodopaminergic state described in cocaine abusers.

Theses findings in human and animal studies suggest that treatments that target KOR signaling would modulate cocaine-seeking behavior. However, animal studies exploring the effect of KOR agonist or antagonist administration on cocaine self-administration are mixed (for review, see Wee and Koob, 2010; Butelman et al., 2012). Partly, this effect depends on the reinforcement schedule used, doses of drug administered, and timing of the effect, since changes in KOR/DYN have a slow onset (Wee et al., 2009; Knoll et al., 2011). Moreover, the DYN/KOR system appears to play a more significant role in mediating the aversive effects that occur with cocaine exposure.

### Kappa receptor/dynorphin and stressed-induced cocaine-seeking behavior

Animal studies have investigated the relationship between KOR activation and stress-induced cocaine-seeking behavior. DYN is released in response to physical stress in the striatum, amygdala, and hippocampus (Shirayama et al., 2004; Land et al., 2008), and blockade of the KOR reduces the effects of stress on cocaine-seeking behavior. McLaughlin et al. (2003) showed that swim stress and social defeat stress both significantly enhance conditioned place preference (CPP) for cocaine in mice. This effect was blocked by KOR antagonist administration and was not seen in prodynorphin knock-out mice (McLaughlin et al., 2003, 2006). In addition, the administration of a KOR agonist prior to cocaine conditioning was shown to be as effective as stress in potentiating subsequent cocaine-induced CPP (McLaughlin et al., 2006). Beardsley et al. (2005) showed that lever pressing for cocaine is reinstated in rodents following uncontrollable footshock, and that this effect is blocked by the administration of JDTic, a KOR antagonist. Along these same lines, Redila and Chavkin (2008) showed that intermittent foot shock, forced swim, and KOR agonist administration all reinstate cocaine CPP in mice. This effect was blocked with pre-treatment with the KOR antagonist nor-BNI, and did not occur in mice lacking either the KOR or prodynorphin. Carey et al. (2007) also showed that pre-treatment with a KOR antagonist blocked stress-induced reinstatement of cocaine CPP.

These studies show that signaling at the KOR plays a significant role in cocaine-seeking behavior following stress. Recent studies have also shown that DYN signaling and corticotropin releasing factor (CRF) function together to increase the negative reinforcing effects of cocaine (Koob et al., 2004). Land et al. (2008) used a phospho-selective antibody for the activated form of KOR and showed that both physical stress and CRF administration resulted in DYN-dependent activation of the KOR. Valdez et al. (2007) showed that, in monkeys, cocaine-seeking behavior is reinstated by the administration of a KOR agonist, and that this effect is blocked by CRF antagonist administration. KOR agonists stimulate the HPA axis in rodents and humans (Ur et al., 1997; Laorden et al., 2000), and it has previously been reported that KOR activation elicits CRF release (Nikolarakis et al., 1986; Song and Takemori, 1992) and vice-versa (Land et al., 2008).

Studies in human cocaine abusers have also shown that stress increases the risk of drug abuse and relapse (De La Garza et al., 2009). The pharmacological or psychological activation of the hypothalamic pituitary adrenal axis has been shown to increase craving in addition to the probability of increased cocaine use (Elman et al., 2003; Shoptaw et al., 2004; Elman and Lukas, 2005). Sinha and colleagues have shown that stress imagery increases anxiety and craving for cocaine (Sinha et al., 1999, 2006; Fox et al., 2006). Importantly, this group has also shown that stress-induced cocaine craving is associated with a shorter time to relapse in cocaine-dependent subjects following discharge from inpatient treatment (Sinha et al., 2006). To date, the imaging studies in addiction have not focused on stress-induced reinstatement of cocaine-seeking behavior, and future research should focus on the role of dopamine and KOR signaling and stress.

Thus, DYN/KOR signaling appears to play a crucial role in reinstating drug-seeking behavior by mediating the negative effects associated with drug cessation and stress-induced drug taking (Koob and Le Moal, 2008; Muschamp and Carlezon, 2013).

## Conclusion

The data presented here suggest that blunted striatal dopamine release measured with imaging in cocaine dependence may be associated with an upregulation of DYN. Acting at the KOR of the dopamine terminals, KOR activation would be expected to produce a decrease in striatal dopamine release. Post-mortem studies in cocaine abusers and animal studies show that both KOR and DYN are upregulated following chronic cocaine exposure, and that this effect is long lasting (Spangler et al., 1993, 1996). In addition, the imaging studies in cocaine abusers show that blunted dopamine release is associated with an increased risk of relapse while animal studies show that activation of the KOR increases cocaine self-administration. However, studies have not been conducted measuring KOR and striatal dopamine signaling in human cocaine abusers concurrently. Thus, future studies imaging the KOR in cocaine abusers and correlating their level directly with dopamine transmission, and with relevant clinical outcomes, is needed.

Chronic cocaine exposure induces CREB phosphorylation and changes in gene expression, which increase expression of prodynorphin mRNA in the nucleus accumbens in addition to other factors. As described above, excessive DYN signaling results in a decrease in extracellular dopamine release, which has been shown in the imaging studies of human cocaine abusers. These findings suggest that increasing signaling at the dopamine receptors may be an appropriate treatment approach, but clinical studies using dopamine agonists have not shown efficacy (Amato et al., 2011). Thus, pharmacologic manipulations that increase endogenous dopamine may be of use, particularly since imaging studies show that intact dopamine signaling is predictive of a positive treatment response. The data reviewed here suggest that KOR antagonists would be expected to counteract the effects of DYN upregulation and may restore pre-synaptic dopamine release. In addition, KOR antagonists have very limited, if any, nervous system side effects (Kreek et al., 2012) and block stress-induced cocaine self-administration in animal studies. Together, these findings suggest that KOR antagonists may provide an important avenue for future treatment development for cocaine addiction (Muschamp and Carlezon, 2013).

## Conflict of Interest Statement

The authors declare that the research was conducted in the absence of any commercial or financial relationships that could be construed as a potential conflict of interest.
